# Integrating condensates into protein lifespan – From birth to death

**DOI:** 10.1016/j.jbc.2026.113271

**Published:** 2026-06-19

**Authors:** Chloe A. Langridge, Emily M. Sontag

**Affiliations:** Department of Biological Sciences, Marquette University, Milwaukee, Wisconsin, USA

**Keywords:** aging, misfolded protein clearance, molecular chaperones, neurodegenerative diseases, phase separation, protein aggregation, protein quality control, proteostasis

## Abstract

Proper protein production is an essential biological process for all known living organisms. The cell depends on a functional protein homeostasis, or proteostasis, network to facilitate proper gene expression, transcription, translation, polypeptide folding, and degradation. Several condensates are involved in the “birth” and maturation of newly synthesized proteins. In conditions of cellular stress, gene expression and protein production are altered, and protein degradation often increases. Under various types of cellular stress, new condensates often form. This review will highlight the condensates that are involved in the protein lifespan. This includes condensates that regulate gene expression, protein translation, sequester misfolded proteins, and those associated with protein degradation. We will summarize what is known about these condensates under unstressed conditions, and how their regulation changes under stressed conditions. Understanding the functions and regulation of condensates during the protein life cycle will be critical to determining how things go awry in disease.

Accurate protein synthesis and degradation are essential for cell health and survival (1, 2). The spatial localization of these processes is important for efficient protein production and turnover. To maximize the efficiency of protein production and degradation, the cell utilizes liquid-liquid phase separated droplets called condensates. Although several condensates are stress-induced, there are a subset of condensates that do not rely on environmental stressors. Condensates like histone locus bodies (HLBs) and processing bodies (P-bodies) act as hubs to regulate protein expression by altering epigenetic expression (3, 4, 5) or preventing the translation of mRNAs (6, 7, 8). Although epigenetic expression and mRNA translation can be greatly altered by stress, it is important to note that some condensates function without environmental stressors.

Environmental stressors trigger the formation of stress-induced compartments often associated with the sequestration of misfolded proteins. These compartments have been found in the nucleus and cytoplasm of *Saccharomyces cerevisiae*, *Caenorhabditis elegans*, and mammalian cells (9, 10). Although the exact purpose of these compartments remains unknown, studies have linked sequestration of misfolded proteins to degradation through the ubiquitin proteasome system (UPS) (11, 12) or autophagy (13, 14, 15, 16). Other compartments appear to be a site of terminal sequestration of misfolded proteins to sequester them away from the cellular environment. This prevents transmission of misfolded proteins to daughter cells during division by facilitating asymmetric inheritance (17, 18). This review will cover the function and regulation of condensates that control protein production, quality control, and degradation under unstressed conditions as well as those specifically induced during cellular stress.

## Condensates involved in gene regulation

**Figure 1:**
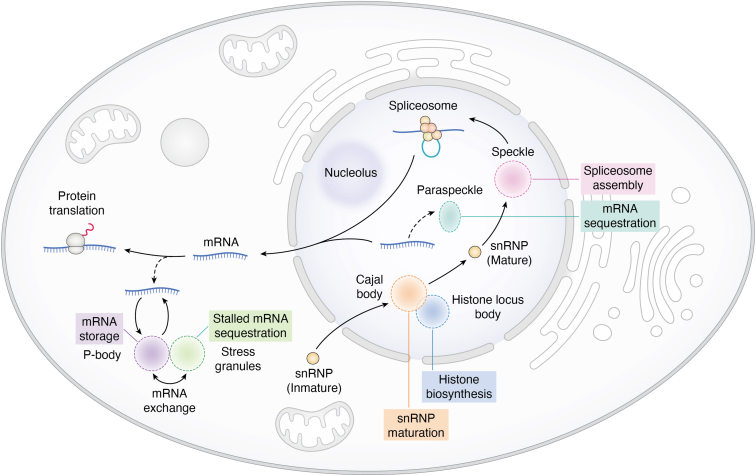
**Condensates associated with gene regulation and protein expression.** In the nucleus, Cajal bodies (CBs) modify snRNPs. Mature snRNPs exit CBs and may enter speckles promoting spliceosome assembly. Other condensates in the nucleus include paraspeckles, which sequester mRNAs in the nucleus, histone locus bodies (HLBs) sites of histone biosynthesis, and the nucleolus. In the cytoplasm, mRNAs may be sequestered in stress granules when translation is stalled or P-bodies to prevent the translation of mRNAs. snRNP, small nuclear ribonucleoprotein.

The nucleus contains many proteins that are intrinsically disordered or contain low complexity sequences which are prone to misfolding and aggregation (13, 19, 20). As such, there are many different membraneless compartments within the nucleus that sequester misfolded proteins, RNA, and RNA-binding proteins as well (Fig. 1). The current view in the field is that nuclear compartments facilitate macromolecule biogenesis by clustering target genes, transcription factors, and RNA processing enzymes (21).

### Nucleolus

The largest and most well-known nuclear body is the nucleolus. Although the nucleolus is best known for its role in ribosome synthesis and processing (22), it serves an important role in transcriptional regulation as well (23). The interaction between the nucleolus and chromatin at nucleolar-associated domains facilitates the recruitment of methyltransferase G9a to add repressive histone modifications (24, 25). It has been proposed that the nucleolus sequesters chromatin from the transcriptionally active environment to aid in gene silencing, because nucleolar interaction correlates with transcriptional repression (26, 27). In addition, the nucleolus contacts the tightly packed, transcriptionally silent heterochromatin rather than the less condensed, transcriptionally active euchromatin (27, 28, 29). In line with this, Peng *et al*. found that all nucleolar-associated domains were transcriptionally inactive (27). Because the nucleolus consists of many different layers of liquid-like phases that do not intermix (30), further work is necessary to distinguish the structural interaction between nucleolar-associated domain and nucleoli.

During stress, the nucleolus undergoes a dramatic reorganization including segregation and/or fragmentation (31). Misfolded proteins enter the outer liquid-like phase of the nucleolus and molecular chaperones such as Hsp70 are recruited to prevent aggregation and promote extraction and protein refolding or clearance after the stress is alleviated (30). Disruption of the outer layer leads to the formation of protein aggregates, highlighting the role of this liquid-like layer in maintaining proteostasis. Furthermore, prolonged stress causes the nucleolus to shift from liquid-like to a solid state, thus preventing protein quality control (PQC) (30, 32). Destabilized nucleoli are also associated with aging and disease. For example, the E3 ubiquitin ligase Mdm2 gets sequestered in the nucleolus during stress where it can no longer bind and target p53 for degradation, leading to activation of p53, cell cycle arrest, and apoptosis (33). Furthermore, nucleolar fragmentation accelerates aging in yeast and is tied to premature aging in humans, highlighting the importance of understanding nucleolar biology (34).

### Histone locus bodies

Histones are fundamental for the epigenetic modification and transcriptional regulation of the genome (35, 36). Modifications of histones like methylation and ubiquitination can promote or repress the expression of specific genes (37, 38). Histones synthesized by HLBs are bound to DNA in the nucleus where they regulate gene expression (39, 40). Different posttranscriptional modifications on histones can either activate or repress gene expression by promoting euchromatin or heterochromatin formation, respectively (41). Histone modifications are crucial under stress. For example, blocking the acetylation of H3K56 increases susceptibility of yeast cells to DNA damage agents such as methyl methanesulfonate and camptothecin (42, 43).

HLBs greatly contribute to the synthesis of histones that can be modified and contribute to transcriptional regulation (44, 45). The formation of HLBs requires the NPAT protein to bind to the C terminus of the pre-mRNA processing factor FLASH, as well as the oligomerization of both proteins (46, 47). In the S phase of replication, NPAT is phosphorylated by Cyclin E, activating HLBs by reorganization and recruitment of the histone cleavage complex (48). Histone mRNA is synthesized in active HLBs (49, 50). Histone transcription in HLBs is inhibited by the YARP protein. Because YARP binds NPAT similarly to FLASH in the formation of the histone cleavage complex (51), it is possible that YARP negatively regulates histone mRNA synthesis by displacing the synthesis complex. Future studies will elucidate the exact mechanism of the regulation of histone mRNA synthesis by YARP.

### Cajal bodies

In physical proximity to HLBs are Cajal bodies, condensates proposed to serve as sites for assembly, modification, and maturation of small nuclear ribonucleoproteins (snRNPs) (45, 52). Because HLBs and Cajal bodies are physically associated with each other and both contain high concentrations of coilin, they were initially difficult to distinguish from one another (45, 53). However, HLBs contain the U7 snRNP and are associated with the histone gene loci whereas Cajal bodies are not (49, 54). Interestingly, the phosphorylation state of coilin regulates Cajal bodies as they form under decreased levels of coilin phosphorylation and disassemble under increased levels of coilin phosphorylation (55). Cajal body formation increases snRNP biogenesis (56, 57). Immature spliceosomal snRNPs are localized to Cajal bodies where the snRNA is modified by small RNAs within Cajal bodies (58, 59, 60).

The presence of RNA is necessary for coilin oligomerization, and oligomerized coilin in complex with snRNPs is required for Cajal body formation (61). It has also been shown that the posttranslational modification SUMOylation of coilin is required for Cajal body cohesion. When the SUMOylation of coilin is disrupted, more Cajal bodies form, but they are much smaller than when coilin is SUMOylated, suggesting the redistribution of Cajal body components associated with the stress response (62). SUMOylation may work in synergy with phosphorylation to regulate Cajal body dynamics, but further investigation is required. Perhaps, coilin SUMOylation promotes its oligomerization, leading to Cajal body assembly and stability while hyperphosphorylation promotes disassembly.

### Speckles

Once mature snRNPs exit Cajal bodies, they may move to spliceosomal storage and assembly condensates called speckles (63). Speckles localize near active transcription sites (64) and components can be recruited from speckles to sites of transcription. Researchers suggest speckles store splicing factors until they are needed, but splicing itself does not occur within the speckles (65, 66, 67). Interestingly, under ribotoxic stress, Sung *et al*. found an increase in the recruitment of factors required for splice-site recognition (66). In addition to pre-mRNA splicing factors like snRNPs and serine/arginine rich proteins (68), speckles contain kinases and phosphatases known for modifying splicing machinery (69). It is possible that splicing machinery is posttranslationally modified in speckles before it is recruited to active transcription sites. While the precise mechanism of speckles formation is not yet understood, it does require the SON and SRRM2 proteins. It has been proposed that SON and SRRM2 act as scaffolds to recruit RNAs and splicing factors to drive condensate formation (69, 70).

### Paraspeckles

Paraspeckles are stress-induced, irregularly shaped condensates located adjacent to speckles (65). For paraspeckles to form, proteins NONO and SFPQ bind to the long noncoding RNA NEAT1 and drive condensate assembly. NONO and SFPQ both contribute to paraspeckle stability and are required for paraspeckle formation (72, 73). Shelkovnikova *et al*. found paraspeckle formation correlates with abnormal micro-RNA (miRNA) levels (74). However, a recent study from the Duchaine lab demonstrated SFPQ represses transcriptional elongation of miRNA in a paraspeckle independent manner (75). Because Shelkovnikova *et al*. focused primarily on miRNA dysregulation in the context of ALS, it’s possible that SFPQ acts as the first line of defense in miRNA regulation, and paraspeckle formation comes later. Further work is required to tease this apart.

Paraspeckles act as regulators of gene expression under stress by sequestering specific mRNAs and preventing their translation (71, 76). Specific mRNAs sequestered by paraspeckles form hairpins in their 3′ UTR due to inverted repeated Alu elements (77). A 15-nucleotide tandem repeat in the 3′ UTR sequence of paraspeckle localized mRNA Calr, which is necessary for the retention of the mRNA into paraspeckles (78). NONO and SFPQ bind the mRNA and anchor it to the paraspeckle, preventing its export to the cytoplasm and subsequent translation (79, 80). mRNAs sequestered by paraspeckles often have inverted repeated Alu elements. Perhaps, there are different cellular stress conditions that promote paraspeckles to sequester mRNAs with Alu elements that have a specific function.

## Condensates that regulate translation

The cytoplasm is extremely complex and acts as an organelle itself, rather than just a liquid meant to fill the cell. It is anywhere from 2 to 6 times more viscous than water and should be thought of as a gelatin-like consistency rather than a watery liquid (81). This gel-like consistency keeps organelles and other cellular components tethered in place. When cellular components do move throughout the cytoplasm, it is through less viscous regions (82). In addition to membrane encapsulated organelles, condensates help organize the cytoplasm. Condensates can regulate protein synthesis in a manner like those that regulate gene expression (83).

### Processing-bodies

Although P-bodies contain mRNAs and proteins involved in translational repression and 5′ to 3′ decay (84, 85), their role in each process is more heavily debated. Under an initial hypothesis, 5′ to 3′ mRNA decay occurs within P-bodies (86, 87). However, several studies have demonstrated P-bodies are not essential for mRNA decay (88, 89). It is possible mRNA exits the translation cycle before entering P-bodies. Interestingly, it was demonstrated mRNA can exit P-bodies and be subsequently translated (90). Although P-bodies are not essential for mRNA decay, they may shield mRNAs from translation machinery. It is also possible mRNA decay in P-bodies may be kinetically advantageous as proposed by Eulalio *et al*. (89).

Like nuclear condensates, P-body formation is highly dependent on posttranslational modifications. In response to cAMP stimulation, the E3 RING ligase praja2 ubiquitinates DDX6, which then interacts with mRNA to promote P-body formation (91). Another P-body protein DCP1A must be phosphorylated to interact with DCP2, and DCP2 must be phosphorylated to localize to P-bodies (91). However, hyperphosphorylation of DCP1A causes disassembly of P-bodies during cell division (92). Interestingly, DCP1A is also phosphorylated under oxidative stress induced by arsenate treatment and hyperosmotic stress (93). Modification of DCP1A is necessary for regulating mRNA turnover under stress (94) further highlighting that while P-bodies are advantageous under certain cellular conditions, they are not advantageous under others.

### Stress granules

Stress granule formation is dependent on stalled translation initiation, a symptom of severe cellular stresses like heat stress and oxidative stress (95). Within stress granules are mRNAs and RNA binding proteins, but unlike P-bodies, stress granules also contain translation initiation machinery (96). Stress granules form to protect translation machinery and enable translation to continue once the stress is alleviated (95, 97). However, some models suggest selective translation can occur within stress granules. Single-molecule imaging by Mateju *et al*. finds that 5′ UTR of ATF4 can be translated when associated with stress granules (98). It is possible that because ATF4 translation increases under stress, stress granules may selectively translate a subset of mRNAs required for the cellular stress response, a model further supported by work from Smith *et al*. (99).

P-bodies cluster around stress granules when stress granule formation is induced. P-bodies “dock” to stress granules by making short contact with them, but both condensates are distinct and eventually separate (100). When the P-body/stress granule protein TTP is phosphorylated, P-bodies no longer dock around stress granules. Researchers have proposed a model in which mRNAs are exchanged between P-bodies and stress granules during docking. (101, 102, 103). It is possible mRNA exchange between P-bodies and stress granules occurs to increase mRNA decay. Because stress granules translate mRNAs required for the cellular stress response but not others, it is possible that stress granules translate mRNAs like ATF4 that are advantageous for stress and transfer other mRNAs to P-bodies to facilitate mRNA decay.

### Amyloid bodies

Unlike several of the dynamic condensates listed above, amyloid bodies (A-bodies) are defined as static and solid-like condensates that contain regulatory ribosomal proteins and translation factors in mammalian cells (104). Theodoridis *et al*. found A-bodies form under acidosis or heat shock conditions and propose that A-bodies serve as sites for the relocation of cytosolic translation factors during stress. They find the formation of A-bodies to be dependent on stress-induced ribosomal intergenic spacer noncoding RNA and argue A-body formation supports nuclear translation under stress. However, the Audas lab found that the components of A-bodies change depending on the stress condition by which they are formed (105). For example, APC2, a tumor suppressor, only localizes to A-bodies under transcriptional-proteotoxic stress while FEN1, a critical protein in DNA replication, only localizes to A-bodies following heat shock. Notably, there are universal A-body proteins that localize to the condensates under all stresses (105). Interestingly, disease-associated proteins such as mutant forms of SOD1 and FUS also localize to A-bodies. However, it should be noted that A-bodies were still induced by the stressors listed above (106) indicating that the disease-associated proteins may not be forming A-bodies alone.

## Compartments for misfolded protein storage

**Figure 2:**
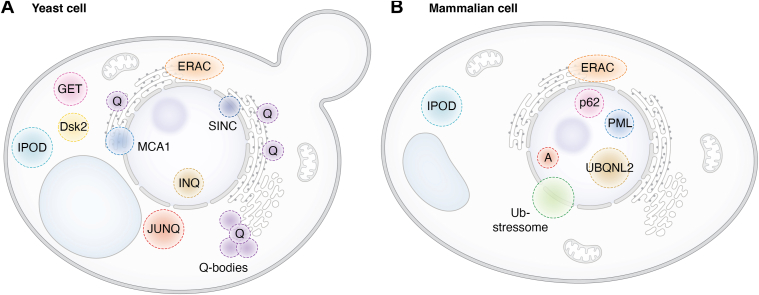
**Yeast and mammalian cell compartments associated with protein stress.***A*, in yeast cells, the intranuclear quality control (INQ) and storage of improperly assembled nuclear pore complexes (SINC) compartment forms in the nucleus. In the cytoplasm, Q-bodies, juxtanuclear quality control (JUNQ), insoluble protein deposit (IPOD), Get bodies, and Dsk2 condensates form. ER-associated compartments (ERACs) form in the ER. *B*, in mammalian cells, promyelocytic leukemia (PML) bodies, p62 nuclear condensates, amyloid *A* bodies, and UBQLN2 condensates form in the nucleus. The IPOD forms in the cytosol. It is unclear if the ubstressome/MCA1 condensates form in the nucleus or cytosol. Q-body, quality-control body; IPOD, insoluble protein deposit.

Proteins that are translated into polypeptides are subsequently folded into native structures. The native protein structure is essential for ensuring the function of the protein. Cellular stressors like heat stress, oxidative stress, and DNA damage can alter the structure of the protein into a misfolded state in which the protein is folded into a conformation different from its native structure. Misfolded monomeric proteins can accumulate into misfolded oligomers and aggregates associated with cellular toxicity (107). They can also form compartments in the nucleus, cytoplasm, and ER to facilitate degradation of the misfolded proteins. Although several compartments do form in mammalian systems, this section primarily focuses on compartments in yeast, which are the most well-characterized (Fig. 2). For more detail on the conservation of the compartments discussed below see (Table 1).

**Table 1:** Summary of condensates discussed

Condensate	Location	Function	Conservation	References
Nucleolus	Nucleus	Ribosome synthesis/processing and transcriptional regulation	Eukaryotes	(21, 22)
Histone locus bodies	Nucleus	Histone synthesis	*Drosophila melanogaster,* mammals	(3, 46, 47)
Cajal bodies	Nucleus	snRNP assembly, modification, and maturation	*Drosophila melanogaster*, mammals, *Xenopus, Arabidopsis thaliana*	(45, 52, 170, 171)
Speckles	Nucleus	Spliceosome storage and assembly	*Drosophila melanogaster*, mammals, *Arabidopsis thaliana*	(63, 69, 172, 173)
Paraspeckles	Nucleus	mRNA sequestration	Mammals	(72, 77)
P-bodies	Cytosol	mRNA decay	Mammals, *Drosophila melanogaster*, *Arabidopsis thaliana, Saccharomyces cerevisiae, Caenorhabditis elegans*	(85, 86, 90, 174, 175, 176, 177)
Stress granules	Cytosol	Protect translation machinery	*Drosophila melanogaster,* mammals, *Arabidopsis thaliana, Saccharomyces cerevisiae, Caenorhabditis elegans*	(97, 98, 99, 101, 177, 178, 179, 180)
Amyloid bodies	Nucleus	Relocation of cytosolic translation machinery	*Mammals*	(104, 105, 106)
PML bodies	Nucleus	Sequesters misfolded proteins	Mammals	(112, 120, 121)
INQ	Nucleus	Sequesters nuclear misfolded proteins	*Saccharomyces cerevisiae*	(9, 13, 122)
SINC	Nucleus	Sequesters nuclear pore complex components	*Saccharomyces cerevisiae*	(123, 124)
Q-bodies	Cytosol	Sequesters cytosolic misfolded proteins	*Saccharomyces cerevisiae*	(13, 125, 126)
JUNQ	Cytosol	Sequesters soluble, cytosolic misfolded proteins	*Saccharomyces cerevisiae*	(12, 13)
IPOD	Cytosol	Sequesters insoluble, cytosolic misfolded proteins	Mammals, *Saccharomyces cerevisiae*	(12, 15)
Get bodies	Cytosol	Protects GET pathway chaperones	*Saccharomyces cerevisiae*	(150, 155)
ERAC	ER	Sequester ER misfolded proteins	Mammals, *Saccharomyces cerevisiae*	(149, 181)
p62 nuclear condensates	Nucleus	Recruits UPS components to misfolded proteins	Mammals	(160, 161)
ubstressome	Unclear	Reduces excessive ubiquitin chains	Mammals, Mca1 condensates in *Saccharomyces cerevisiae*	(164)
UBQLN2 condensates	Nucleus	Sequesters ubiquitinated substrates	Mammals, Dsk2 condensates in *Saccharomyces cerevisiae*	(165, 166, 167, 168, 169)

The function, location, and conservation amongst organisms is highlighted.

### Nuclear compartments

Stresses like DNA damage can lead to errors in transcription, translation, and protein misfolding (108). To prevent protein aggregation in the nucleus, misfolded proteins are sequestered into spatially distinct compartments proposed to serve as alternative clearance mechanisms to prevent senescence and apoptotic mechanisms from occurring (13, 108, 109).

#### Promyelocytic leukemia bodies

Promyelocytic leukemia (PML) bodies depend on the PML protein for formation (109). Because PML bodies depend on the PML protein, they are specific to mammalian systems. During stress, the PML protein is SUMOylated which promotes the interaction between PML and other SUMOylated proteins at PML’s SUMO binding motif (110). This process drives the formation of a spherical shell that encloses many different types of unrelated proteins (111). This variety of proteins has led to difficulty in identifying the function of PML bodies. PML bodies have been implicated in biological processes as diverse as senescence and stemness, to viral infections, and stress responses—particularly the oxidative stress response (111, 112, 113). There is growing recognition that PML bodies may be indicators of DNA damage (114, 115) because PML body concentrations increase as genotoxic stress increases (115, 116). Regions concentrated with PML bodies can be attributed to regions of damage, especially double-stranded DNA breakage, yet it remains unclear if they are directly involved in DNA repair.

A well-known symptom of DNA damage is protein misfolding and the accumulation of misfolded proteins into aggregates. Recent work by Guo *et al*. demonstrated that the SUMOylation of the PML protein enables it to physically interact with misfolded proteins. Using Atxn1 82Q as a substrate, they showed that the presence of PML protein promotes SUMOylation of Atxn1 and proposed that Atxn1 is degraded by the proteasome in an RNF4-dependent manner (117). It was also shown that TDP-43 enters PML bodies and misfolds upon prolonged stress. They propose that PML bodies serve as deposition sites to prevent the degradation of proteins (118). It is possible proteins initially enter PML bodies to be protected from degradation, but under severe or prolonged stress, they misfold and are subsequently targeted for degradation by the proteasome.

#### Intranuclear quality control compartment

In budding yeast, misfolded proteins localize to the intranuclear quality control (INQ) compartment under DNA damage (9, 13, 124). Like PML bodies, the INQ is comprised of misfolded proteins in the nucleus (119). Proteins associated with chromatin remodeling, transcription, and replication fork structure cleavage relocalize to the INQ under proteotoxic stress (20). Interestingly, the INQ seems to selectively recruit these factors. Stirling *et al*. found the splicing factor Hsh155 disassembles from its partners and localizes to the INQ, but its partners do not (125). Substrates of the INQ do not have strong functional commonalities, but several do have at least one WD40 domain. Deletion of this domain from the INQ substrate Cmr1 inhibits its localization to the INQ (20, 126). More work is required to determine how the WD40 domain contributes to liquid-liquid phase seperation and compartment formation.

The INQ can move in parallel with another misfolded compartment in the cytosol called the juxtanuclear quality control compartment (JUNQ) near the nucleus-vacuole junction (13). When clearance is impaired, the INQ forms a nuclear bud and is cleaved in an exocytotic fashion. Although the specifics of this cleavage mechanism are still not understood, there is a general hypothesis to explain this phenomenon. ESCRT-III, an endosomal sorting complex required for transport, collaborates with ATPase Vps4 to constrict membrane regions enabling budding scission. Homing between the INQ and the JUNQ stops after this release. The cleaved INQ is transported to the vacuole and degraded. Interestingly, Samant *et al*. found nuclear misfolded proteins can be ubiquitinated and targeted for degradation by the proteasome in a Dsk2-dependent manner (122). Further work is required to determine when it is more advantageous to shuttle to the INQ or to the proteasome by Dsk2.

#### Storage of improperly assembled nuclear pore complexes compartment

The storage of improperly assembled nuclear pore complexes (SINC) compartment resides in the nuclear envelope and sequesters damaged or misassembled nuclear pore complexes in yeast (123). Like the INQ, the SINC is related to endosomal sorting complexes required for transport (ESCRT)-III machinery. Under normal conditions, Webster *et al*. propose ESCRT-III and Vps4 are required for recognizing and removing defective intermediates of the nuclear pore complex prior to mature assembly. These defective intermediates may then be targeted for clearance mechanisms. If the defective intermediates are fully assembled into a nuclear pore complex, the entire complex is targeted to the SINC. It is likely Chm7 recruits nuclear pore intermediates to the SINC because disrupting the assembly of nuclear pore complexes leads to the accumulation of the ESCRT-II/III protein Chm7 at the nuclear envelope (124). More work is required to understand the mechanism that leads to SINC formation, including any other sorting factors or chaperones involved in recognition of defective nuclear pores.

### Cytoplasmic compartments

Like in the nucleus, cytosolic misfolded proteins are sequestered into spatially distinct compartments (9, 12, 13, 14, 125). Cytosolic compartments are specific to the type of protein misfolding. For example, the JUNQ sequesters soluble protein while the insoluble protein deposit (IPOD) sequesters insoluble proteins known to form fibrillar aggregates like the mutant Huntingtin (mHtt) protein associated with Huntington’s disease (12).

#### Q-bodies

Q-bodies are small, dynamic inclusions in yeast that form early after heat stress. Q-bodies may dissolve early on or become sequestered in the more terminal compartments like JUNQ and IPOD as discussed below (13, 125). Interestingly, Q-body formation appears to correlate with P-body formation. When the Holt lab induced Q-body formation in cells lacking the P-body protein Edc3, less Q-bodies formed in cells (126). Q-body formation is dependent on the small heat shock protein Hsp42 as they do not form when *HSP42* is deleted (125, 127). The N-terminal region of Hsp42 contains a prion-like domain and an intrinsically disordered region (IDR). Interestingly, the prion-like domain is essential for client binding and enhances the oligomerization of Hsp42 (127). Deleting the adjacent IDR increases the number of inclusions. The IDR is thought to act as a regulatory domain of client binding to Hsp42 as deleting the IDR reduced fitness and increased Hsp42 client binding (127). It would be interesting to determine if the chaperones required for Q-body maturation also bind to the Hsp42 IDR. Perhaps the IDR acts as a hand-off site rather than a regulatory domain as IDRs are often associated with chaperone interactions (128).

The association of Q-bodies with cortical and perinuclear endoplasmic reticulum (ER) is dependent on the Hsp40s Ydj1 and Hlj1 (125). The C-terminal farnesyl group of Ydj1 allows for insertion into the ER membrane (129, 130). Deleting Ydj1 or mutating its farnesyl group partially inhibits the association of Q-bodies with ER (125). Hlj1 contains a C-terminal hydrophobic “tail” that anchors it to the ER membrane (131). Deleting Hlj1 also partially inhibits Q-body association with ER (125). It is hypothesized that Q-bodies use Ydj1 and Hlj1 to move along the fluid ER membrane (125) and individual Q-bodies coalescence into the JUNQ. However, we have demonstrated that the JUNQ is composed of individual Q-bodies rather than a single large inclusion (13, 132). Perhaps Q-bodies utilize ER-associated Hsp40s for mobility where they eventually cluster into the JUNQ.

It remains unclear why Q-bodies are concentrated with chaperones and what the role chaperones play in the proteostasis process. Deleting Hsp90 delays Q-body coalescence and impairs clearance, but how this occurs remains unknown (125). Hsp90 plays a key role in refolding (133) and similarly, Hsp70 contributes to refolding and proteasome targeting (134). However, Escusa *et al*. suggest Hsp70 plays an essential role in targeting inclusions to Q-bodies because Hsp70 deletion resulted in impaired clearance and the formation of one predominant inclusion (125). Because refolding factors are present in Q-bodies (17, 125) and fluorescence loss in photobleaching (FLIP) analysis demonstrates high exchange rates with the cytosolic pool (127), there may be situations where refolding is advantageous for cellular survival. Because Q-bodies are enriched in Hsp70, Hsp90, and Hsp104, client proteins may be refolded and selectively released from Q-bodies (17, 125, 135).

#### Juxtanuclear quality control compartment

The JUNQ is a dynamic compartment of soluble, ubiquitinated Q-bodies concentrated near the nucleus-vacuole junction in yeast (12, 13). JUNQ formation is dependent on the targeting factor Btn2 (136, 137). Btn2 was originally thought to recruit chaperones required for dissolution of Q-bodies (137), leading to the resolubilization of Q-body components into the JUNQ. However, we utilized cryogenic X-ray tomography and cryogenic electron tomography to demonstrate that the JUNQ is composed of intact Q-bodies rather than a singular coalesced inclusion, suggesting dissolution is not required for JUNQ formation (13, 132). JUNQ formation is modulated by the physical interaction between Btn2 and the Hsp40 Sis1 (136). Although it is known that Sis1 binds the IDR of Btn2, it is unclear how this interaction contributes to JUNQ formation.

JUNQ inclusions recover from a concentrated punctum to a state of diffuse fluorescence, suggesting they are either refolded or cleared (12). However, how this process occurs is largely unknown. The JUNQ may selectively release misfolded protein clients to the proteasome or clients rejected by the proteasome may be targeted to the JUNQ. In line with this, JUNQ formation is predominantly dependent on proteasomal stress, it localizes close to proteasomes, and FLIP analysis shows high exchange rates with the cytosolic pool. If chaperones facilitate refolding within inclusions, refolded clients may be released in the cytoplasmic pool. Hsp104 and Hsp70 localize to JUNQ inclusions, suggesting misfolded proteins can be threaded and unfolded by Hsp104 and then subsequently refolded. Because the JUNQ concentrates ubiquitinated Q-bodies and ubiquitination is required for clients to be canonically degraded by the proteasome (138), clients may be selectively released from the JUNQ and targeted to the proteasome. Alternatively, due to the JUNQ’s localization to the nucleus-vacuole junction, the JUNQ may be degraded by the vacuole (13). This process was recently uncovered in the INQ. Because the INQ moves synchronously with the JUNQ at the nuclear vacuolar junction up until INQ clearance through the nuclear vacuolar junction, the JUNQ may also be targeted to the vacuole.

#### Insoluble protein deposit

The IPOD sequesters proteins that are insoluble in yeast and mammalian cells (12); however, the majority of the follow- up studies have been performed in yeast. The perivacuolar IPOD is located adjacent to the preautophagosomal structure (12, 139). Unlike the JUNQ, the IPOD serves as a deposition site for terminally misfolded and amyloid-forming proteins (12, 140). Btn2 and Hsp42 are not required sorting factors as they are for the JUNQ (136, 141). Although Hsp42 is involved in sorting between the IPOD and the JUNQ, one study found that the deletion of *HSP42* did not have a major effect on amyloid trafficking to the IPOD (142). Interestingly, the E3 ligase Ltn1 was found to promote the accumulation of mHtt103QP aggregates to the IPOD. Deletion of Ltn1 increased the number of mHtt103QP aggregates. These aggregates colocalized with actin and disrupted endocytosis (143). It has been hypothesized that amyloid aggregates are transported to the IPOD by moving along actin cables (15). However, another model suggests this is an indirect process where aggregates are recruited to the IPOD by association with Atg9 vesicles (15).

The IPOD itself has been proposed to hitchhike on COPII vesicles to be trafficked to the vacuole for degradation (144). There is little confirmation as to how this process occurs, but IPOD trafficking by different components of the endo-lysosomal degradation pathway appears to be the most accepted. Babazadeh *et al*. demonstrate that the IPOD is transported from the ER to the Golgi by associating with COPII vesicles (144). From this point, the authors show that the IPOD associates with the CORVET complex to transport from the Golgi to an early endosome. Transport to multivesicular bodies also includes CORVET with the addition of the HOPS complex. Because HOPS is also associated with the IPOD’s delivery to the vacuole, Babazadeh *et al*. hypothesize CORVET delivers the IPOD to multivesicular bodies and hands the IPOD off to HOPS. HOPS can then transport the IPOD to the vacuole. This hitchhiking model illustrates that the IPOD is not formed at the vacuole as previously suggested (12). Instead, the IPOD is formed in the cytoplasm and transported to the vacuole. It is possible that the IPOD’s proximity to the vacuole more efficiently allows for its vacuolar degradation once it is full of the insoluble protein aggregates.

### Compartments associated with the endoplasmic reticulum

The ER is responsible for a major fraction of protein synthesis (145) and it is no surprise that the ER has its own quality control system. Under conditions of cellular stress, the sheets of the ER physically expand (146). Because the sheets are the preferred site of protein synthesis, folding, and posttranslational modifications, it is likely that expanding the ER surface area is advantageous for managing stress. During ER stress, ER-associated degradation (ERAD) and the unfolded protein response (UPR) are initiated (147, 148). In addition to having its own stress pathways, the ER forms compartments that prevent misfolded proteins from clogging synthesis or transport pathways to protect transport machinery important for cellular fitness (149, 150).

#### Get bodies

As mentioned in previous sections, ER-associated chaperones are necessary for managing the cellular stress response. In yeast, the insertion of tail-anchored (TA) proteins like the Q-body chaperone Hlj1 into the ER membrane is dependent on the guided entry of TA proteins (GET) pathway (151, 152). At the 80S ribosome, Hsp70 binds a TA protein before its transmembrane domain (TMD) is exposed to the cytosol (153). Hsp70 then binds to the tetratricopeptide repeat (TPR) domain of Sgt2 to promote effective association of TA proteins with the C terminus of Sgt2. The Sgt2 dimer will either close to clamp down on the client and initiate the GET pathway or remain open the TA protein will not enter the GET pathway (154).

Under ATP depleting stress conditions like glucose starvation, Sgt2 facilitates the formation of cytosolic Get bodies including GET pathway machinery but excluding TA proteins (150, 155). Recently, the GET pathway chaperone Get3 was found to moonlight as a holdase under oxidative stress, another ATP depleting condition (163, 164). During oxidative stress, the cysteines of Get3 are oxidized and Get3 loses nucleotide binding ability and partially unfolds. This induces a conformational change in which the TA-binding domain of Get3 is buried, leading to Get3 oligomerization. Get3 also forms inclusions during oxidative stress, but it is unclear if these are the same Get bodies that form under glucose starvation. However, the chaperone function of Get3 is conserved in its mammalian homolog (156). It is possible the Get3 holdase function may be an important tool in understanding the cellular response to oxidative stress.

#### ER-associated compartments

Get bodies sequester the TA protein insertion machinery associated with the ER; however, proteins that misfold in the ER are sequestered into separate compartments termed ER-associated compartments (ERACs) (149). ERACs are known to form in yeast and mammalian cells (16, 157). While many compartments discussed above exist in the nucleoplasm or cytoplasm, ERACs are connected to the ER. COPII machinery is responsible for sequestering misfolded ER substrates to ERACs, however, ERAC formation is independent of ER-to-Golgi transport (157). Kakoi *et al*. therefore hypothesize that COPII machinery facilitates ERAC formation independent of its known function in ER-to-Golgi transport. They found the clearance of ERACs is driven by autophagy as the deletion of autophagy factors Atg1 and Atg6 inhibits the clearance of ERACs, but inhibiting the proteasome had no effect on ERAC degradation. Therefore, they proposed that the misfolded proteins that cannot be degraded by the proteasome enter ERACs where they are degraded by autophagy, suggesting a larger ER quality control pathway is occurring. Interestingly, ERAC formation does not induce the unfolded protein response (16). Because the unfolded protein response handles luminal stress, it’s likely that ERAC formation is a result of membrane and cytosolic localized misfolded proteins rather than luminal misfolded proteins.

## Condensates associated with proteasomal degradation

Misfolded proteins are often ubiquitinated and targeted for degradation by the 20S proteasome. Proteasomal degradation is a highly regulated and energetically expensive biological process. Because proteasomal degradation is essential for biological function, there are several condensates that aid in its regulation. For example, when the Dantuma lab blocked stress granule formation, they found a defect in the UPS (158). The authors support a model in which stress granules sequester cytosolic misfolded proteins and prevent them from being clogging the proteasome under stress (158). Several other condensates have recently been characterized in proteasome regulation such as p62 nuclear condensates, ubstressomes, and UBQLN2 condensates as discussed below.

### p62 nuclear condensates

To increase the efficiency of proteasomal degradation, p62 nuclear condensates recruit UPS components and protein substrates in mammalian cells (159, 160). Fu *et al*. first characterized p62 nuclear condensates and found that they form under both unstressed and stressed conditions. They also showed that p62 phase separation increases degradation of nuclear-localized substrates and unincorporated proteasomal subunits (160). If SINC compartments form in mammalian cells as they do in yeast, it would be interesting to understand the relationship between the two compartments. Perhaps there is substrate transfer between SINCs and p62 nuclear condensates like stress granules and P-bodies.

In addition to increasing proteasome efficiency, p62 nuclear condensates are also involved in stabilizing PML bodies (161). The Ciechanover lab demonstrates p62 nuclear condensates interact with PML bodies, but there is no fusion of the condensates or mixing of their materials. While p62 nuclear condensates are not mixing substrates with PML bodies, the authors found the ubiquitin ligase RNF4 is sequestered and degraded by p62 nuclear condensates. As RNF4 normally facilitates the degradation of PML bodies, the authors support a model in which PML bodies are stabilized by p62 nuclear condensates (161). Because PML bodies and stress granules mix materials, it would be interesting to see how p62 nuclear condensates affect the dynamics of stress granules.

### Ubstressome

Excessive ubiquitin chains or chains that accumulate beyond the normal chain length can impair clearance and contribute to protein aggregation (162). Recently, the Zhang lab identified the ubstressome compartment, a caspase-2 (CASP2) dependent condensate in mammalian systems required for reducing excessive ubiquitin chains (163). The authors found CASP2 is required for regulating excessive ubiquitin chains, and that CASP2 is integrated into a condensate with ubiquitin which they have named the ubstressome. In addition, CASP2 binds excessive ubiquitin chains, and they propose CASP2 acts as a deubiquitinase to remove the excessive ubiquitin chains. Finally, the authors show the caspase homolog MCA1 in yeast also forms condensates and is required for the removal of overloaded ubiquitin chains, suggesting that this process may be conserved amongst organisms. Zhang *et al*. then propose a model in which the ubstressome forms in a CASP2- dependent manner after heat shock where CASP2 removes excessive ubiquitin chains from misfolded proteins, thus allowing for efficient substrate degradation by the proteasome upon ubstressome disassembly (163). This newly discovered compartment has lots of outstanding questions, including the dynamics of the condensate. Further work is required to determine if modified clients can exit the ubstressome, or if disassembly of the ubstressome is required.

### UBQLN2 condensates

Several studies have shown that ubiquilins like UBQLN2 can form condensates, and that disease-associated proteins can enter these condensates (164, 165, 166). Work has demonstrated that the phase separation of UBQLN2 condensates depends on the type of polyubiquitin linkage. The Kraut lab has demonstrated the sequestration of ubiquitinated substrates into UBQLN2 condensates protects them from proteasomal degradation and deubiquitination. The authors found K63-linked substrates were more often sequestered into UBQLN2 condensates than K48-linked substrates. Acharya *et al*. demonstrated that the yeast homolog of UBQLN2, Dsk2, also phase separates. The authors found the Sti1 domain of Dsk2 mediates self-assembly and facilitates phase separation. Recently, Onwunma *et al*. further characterized this process and demonstrated Dsk2 placeholders block the hydrophobic groove in the Sti1 domain of Dsk2 from client interaction and instead promote phase separation (167). Both UBQLN2 and Dsk2 form condensates in the nucleus (164, 168, 169).

## Conclusions and perspectives

Condensates play a pivotal role in the lifecycle of a protein. There is still much to learn about the various functions of cellular condensates. There is strong evidence that condensates involved in gene regulation work together, but how PQC compartments work together remains to be uncovered. The biological relevance of the PQC compartments JUNQ and INQ homing in to the same site on the nuclear envelope is still unknown. Perhaps, misfolded proteins can move between the two under specific stressors. There is also overlap in the chaperones associated with several PQC compartments. Why chaperones go to one compartment over another remains to be uncovered. Can misfolded proteins associated with Q-bodies go to ERACs or do they always go to the JUNQ? Is it related to their clearance pathways? In addition, several compartments associated with misfolded proteins have been identified in yeast, but it is unknown how conserved they are amongst other organisms. To fix a broken cell, we must understand what is broken and how it broke. Understanding how compartments work together is crucial for understanding the functionality of proteostasis. Once we understand their function, we can more effectively develop therapeutics for protein misfolding diseases like neurodegenerative diseases and cancers.

## Conflict of interest

The authors declare that they have no conflicts of interest with the contents of this article.
